# Design of Conceptual Model of Psycho‐Social Burden in Infertile Couples Undergoing Assisted Reproductive Treatments: A Study Protocol for a Mixed Method Study

**DOI:** 10.1002/hsr2.70854

**Published:** 2025-05-26

**Authors:** Shekofeh Maleki, Ashraf Kazemi, Hamid Nasiri‐Dehsorkhi, Nafisehsadat Nekuei

**Affiliations:** ^1^ Stdent Reseach Commetee Isfahan University of Medical Sciences Isfahan Iran; ^2^ Nursing and Midwifery Care Research Center, Reproductive Health Department Isfahan University of Medical Sciences Isfahan Iran; ^3^ Isfahan Gastroenterology and Hepatology Research Center Isfahan University of Medical Sciences Isfahan Iran; ^4^ Reproductive Health Department Isfahan University of Medical Sciences Isfahan Iran

**Keywords:** assisted reproduction, burden, conceptual model, infertility, psycho‐social

## Abstract

**Background and Aims:**

The multifaceted consequences of infertility impose a significant psycho‐social burden on the couples involved and necessitate design a valid conceptual model to identify them. Therefore, this study aimed to design of conceptual model of psycho‐social burden in infertile couples undergoing assisted reproductive treatments.

**Methods:**

This study will be conducted in four phases using a mixed method and the sequential exploratory approach. In the first phase, a qualitative study will be carried out through the content analysis approach with the aim of identifying the components of psycho‐social burden in infertile couples who are candidates for assisted reproduction treatment (ART). In the second phase the initial version of the infertility psycho‐social burden questionnaire will be compiled. Face and content validity will be evaluated quantitatively and qualitatively. In the third phase, a cross‐sectional study will be conducted on 150 infertile couples who are candidates for ART in Isfahan, Iran. The construct validity of the prepared questionnaire will be evaluated using exploratory factor analysis with varimax rotation. After psychometrics evaluation of the infertility psycho‐social burden questionnaire, sampling will continue on 150 couples. Participants' mental health will be evaluated using the 28‐item general health questionnaire, and its correlation with the extracted psycho‐social burden components will be evaluated. Based on the results of the third step, in the fourth phase, a conceptual model of psycho‐social burden will be designed and confirmatory factor analysis of the conceptual model will be performed.

**Conclusion:**

Extracted conceptual models will assist psychological counselors, social workers, and psychoanalysts in gaining an in‐depth understanding of the factors affecting the mental health of infertile couples. Moreover, the psychosocial burden of the infertility scale can be presented as a screening tool for couples at risk of psychological issues related to infertility.

## Summary

- The conceptual model of psychosocial burden and a valid tool for assessing psycho‐social infertility burden will be helpful tools for developing mental health programs for infertile couples undergoing treatment.
- The designed conceptual model will be document based model based on the social context of the target population.
- Utilization of the conceptual model and also, designed questionnaire in other societies needs to be evaluated.

## 1 Introduction

Infertility is a global complication with a prevalence of approximately 20% in couples worldwide, with the highest pervasiveness in developing countries [1]. According to global statistics, roughly 21% of Iranian couples need to receive related services due to infertility [2] and suffer from psycho‐social challenges [3].

In the social structures of many societies, reproduction is the goal and criterion for achieving marital satisfaction [4], social acceptance, and confirmation of gender identity [5]. Therefore, infertility in these communities is considered not only a medical problem but also a social challenge with additional adverse effects on the mental health of couples involved in infertility treatments [6]. The social challenges of infertility in these societies take on wider dimensions since it is mainly attributed to women [7], and can be associated with domestic violence [8], social isolation [9], and polygamy [10]. Also, it can reduce the quality of married life in the infertile couples [11].

Studies have shown that the perception of stigma [12] and social pressure [13] are the recognized experiences of infertile couples in these societies. The relationship between the social consequences of infertility and increased psychological disorders such as depression, anxiety, [14] and stress [15] have also been previously reported. The evidence indicates the psycho‐social burden in infertile couples. A psycho‐social burden is a person's mental perception of a pressure that is imposed due to a complication and causes psychological reactions [12].

Enduring the psycho‐social burden of infertility in couples involved with damage to their mental health may also influence infertility treatment success [13]. Perception of the psycho‐social burden increases the couple's effort to conceal the fertility disorder [14] and adds to the stress of infertility treatment [15].

Therefore, the significance of identify sources of stress in infertile couples seeking treatment accounts for the necessity of evaluating the psycho‐social burden of infertility. A valid conceptual model is essential to understand the psychosocial burden of infertility and its relations with the mental health of infertile couples. This model also aids in developing evidence‐based programs to reduce the stress experienced by couples undergoing assisted reproductive treatment and enhance their mental well‐being.

However, a specific conceptual model of the infertility psychosocial burden, based on the social background of traditional societies, has not been designed. In addition, psychological screening and situational analysis, which is mainly conducted in couples seeking assisted reproductive technique (ART), measures psychological disorders such as depression, anxiety and stress as well as infertility‐related social issues, such as the adaptation to infertility [16], infertility stigma [17] and Fertility quality of life (FertiQol). Fertility quality of life tool is the validated tool used as a valid module to evaluate the impact of infertility complications on the emotional, mental‐physical, relational, and social domains [18]. This tool demonstrates acceptable reliability and sensitivity in assessing infertile couples' quality of life. However, it was not developed to evaluate the psychosocial burden constructs or identify social resources that impose psychological burdens on infertile couples. Instead, it focuses on measuring the impact of infertility on various aspects of the infertile couples' quality of life.

However, the evaluations merely focus on some social aspects of infertility and lack the efficiency to comprehensively measure the psycho‐social burden components. While for evaluating the psycho‐social situation analysis and developing mental health programs based on psycho‐social burden, it will be very useful to present a conceptual model.

Therefore, the present study will be conducted to design of conceptual model of psycho‐social burden in infertile couples undergoing assisted reproductive treatments. This conceptual model provides a module that helps counselors, psychologists, psychoanalysts, and social workers of infertility treatment centers in Iran and similar countries gain a deeper understanding of their clients' psychological conditions and provide more efficient counseling programs.

### 1.1 Objectives

- Determining the psychosocial experiences of couples undergoing assisted reproductive treatment in a study with a qualitative approach,
- Design of psychosocial burden questionnaire items,
- Evaluation of the validity and reliability of the developed questionnaire,
- Evaluating the construct validity of the questionnaire,
- Evaluation of relationships between psychosocial burden constructs and general health domains,
- Development of a conceptual model of psychosocial burden of infertility,
- Confirmatory analysis of the developed conceptual model.

### 1.2 Research Questions

RQ1: What themes does the psychosocial burden of infertility include? (Phase 1)

RQ2: How valid and reliable is the psychosocial burden of infertility scale? (Phase 2)

RQ3: What is the relationship between psychosocial burden constructs and the mental health of infertile couples? (Phase 3)

RQ4: What is the appropriate conceptual model of the psychosocial burden of infertility? (Phase 4)

## 2 Methods and Analysis

**Figure 1:**
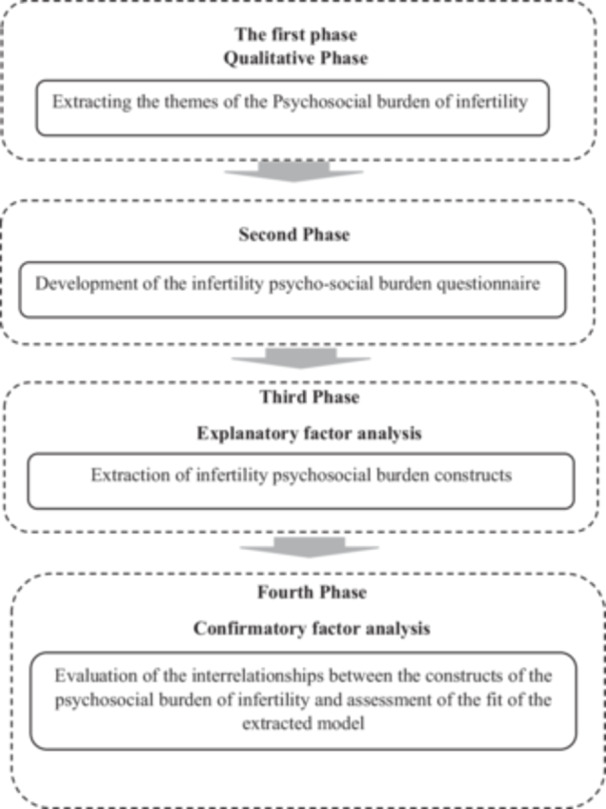
Steps of the study.

This study was designed based on exploratory and confirmatory technique in four phases following a multiphase approach after being approved by the ethics committee of Isfahan University of Medical Sciences (Figure 1). This study will be conducted in two fertility and infertility centers including one private and one public centers in Isfahan, Iran.

The first phase is carried out qualitatively with the aim of evaluating the psycho‐social experience of infertile couples who are candidates for ART. This study phase began in February 2025 and is expected to last 8 months.

The second phase will be based on the results of the first phase with the aim of developing the initial version of the questionnaire. For the third phase, a cross‐sectional study will be designed with the aim of determining the construct and criterion validity of the compiled questionnaire. In the fourth phase, a cross‐sectional study will be conducted to implement a confirmatory analysis of the extracted conceptual model.

All stages of the study will be based on the ethical codes of the Ethics Committee of Isfahan University of Medical Sciences. The interviewer is not a treatment team member; she will introduce herself to participants before inviting them to the study. At each stage, informed consent will be obtained from all participants.

### 2.1 The First Phase: Qualitative Study

The first phase of the study will be conducted with the qualitative content analysis approach, with the aim of explaining the experiences of infertile couples undergoing ART regarding the psycho‐social burden of infertility. The participants of this phase will include two groups including infertile couples who are candidates for ART referred to the fertility and infertility centers, infertility service providers (including physicians, counseling psychologists) and legal experts of the two centers.

The inclusion criteria for infertile couples will include Iranian nationality, Persian language, having rich and complete information and the ability to express it to the researcher, no severe and persistent mental illnesses such as severe depression or bipolar disorder (according to the psychiatrist's opinion and the medical documents), not using donated gamete or the surrogate mother, and no history of successful pregnancy. Inclusion criteria for providers will include a history of providing fertility services for at least 2 years.

### 2.2 Data Collection

Data collection will be performed at this stage using a semi‐structured interview, voice recording using a voice recorder, and field notes. To prepare the interview questions, first, five in‐depth and unstructured interviews will be conducted, and the key interview questions will be formulated based on them. In this interview, participants will be requested to express their experiences, especially in social interactions and social issues they would have faced.

Key questions will be compiled based on this interview, and further interviews will be conducted individually and with couples using these questions. As the interviews proceed, exploratory or probing questions such as ‘Please explain it more’ will be used for deeper understanding.

The first appointment with the participants will be scheduled at the admissions unit of the infertility centers to investigate the inclusion criteria and invite them to participate in the study. Sampling will be performed purposefully with maximum diversity in terms of age, education level, duration of infertility, type and cause of infertility, number of treatment cycles, and gender (1:1 ratio).

After identifying the eligible individuals, the study requirements, objectives, and participation will be explained by one of the researchers. Moreover, obtaining the informed consent as well as the time and location will be identified based on the participants' preferences. Regarding participants' rights, explanations will be provided; they will be informed that participation in the study is entirely voluntary. The interview will be anonymously analyzed, and its results will be reported; reluctance to participate in the study or continue it will not affect the treatment process, and there will be a possibility to withdraw from the study at any time.

It will also be clarified that the recorded voice will only be listened to by the interviewer and the research supervisor in a private environment. The data will be collected manually in cases where the participant will not be content with having their voice audio recorded.

The interviews will be conducted by one of the female researchers, who is a PhD candidate in reproductive health. However, men participants will be interviewed by a man interviewer if they prefer. Before initiating the interview, consent will be obtained from the participant regarding audio recording.

Estimated time for each interview is between 60 and 90 min. If the interview remains incomplete for any reason, the time and location of the subsequent interview will be determined. Moreover, if the researchers need to complete the information during the analysis, a secondary interview appointment will be made with the participant.

#### 2.2.1 Sample Size

The sample size is estimated based on diversity criteria in the study, and at least three participants are considered for three diversity factors [19]. For age diversity, three age groups and education (for both genders), two groups for duration of infertility, cause of infertility, and number of treatment cycles are considered. Accordingly, the sample size was estimated to be at least 54 individuals. Sampling will continue until data saturation. Data saturation is considered the sampling adequacy criterion; the sampling continued until no new inferential codes emerged by continuing the interview and the emergence of repeated inferential codes five times.

#### 2.2.2 Data Analysis

In this phase, the study data will be analyzed using the qualitative conventional content analysis method [20]. To this end, immediately after terminating the interview and at the earliest opportunity, the interviewer will listen to the interview several times and write down its key points.

Following that, the full text of the interviews and field notes will be transcribed verbatim. By repeatedly reading the text, the researcher will attempt to get a deeper understanding of the interview concepts and identify the key parts of the narratives. Inferential codes will be extracted from these parts. Sub‐subcategories, subcategories, and categories will be formed from the inferential codes. Sampling will continue until the previous inferential codes are repeated during the analysis and no new codes are inferred.

#### 2.2.3 Rigor and Trustworthiness

To investigate the accuracy of the data, the criteria proposed by Lincoln and Guba, including credibility, dependability, transferability, and confirmability, will be used [21]. Different methods will be applied to validate the findings, including allocating adequate time and continuous engagement in data collection, combining different data collection methods such as in‐depth interviews and field notes, and recruiting participants with maximum diversity. To confirm the accuracy of the extracted data and codes or modify them, the member check method, and to ensure the findings' adaptability to the participants' statements, the experts' opinions will be used. To increase transferability, the findings of the study will be provided to individuals who did not participate in the study yet have characteristics similar to the participants to judge whether or not the results of the study are similar to their experiences.

### 2.3 The Second Phase: Development of the Infertility Psycho‐Social Burden Questionnaire

In this phase, based on the results of the qualitative section and the literature review, questionnaire items will be designed in the Persian language on a 5‐point Likert scale. The literature review will be conducted in databases, including Scopus, PubMed, Web of Science, Science Direct, Cochrane, ProQuest, CINAHL, IRAN MEDEX, MagIran, SID, and MEDLIB, using the keywords Psycho‐social, burden questionnaire, infertility, couples requesting assisted reproductive technologies, and their Persian equivalents between 2000 and 2024.

#### 2.3.1 Validation of the Questionnaire

At this stage, the face and content validity of the designed questionnaire will be investigated quantitatively and qualitatively with the participation of experts and infertile couples. Furthermore, the internal reliability and repeatability of the questionnaire will be evaluated in a pilot study on 20 eligible infertile couples.

#### 2.3.2 Face Validity

Qualitative and quantitative methods will be used to determine face validity of the questionnaire. To evaluate the qualitative face validity, the prepared questionnaire will be provided to 15 infertile couples seeking ART, and they will be requested to comment on the clarity and comprehensibility of each item's wording and the ease of completing the questionnaire. Furthermore, the questionnaire and the purpose of the study will be provided to 15 experts in the field of instrumentation, psychology, reproductive health, and psychiatry, and they will be requested to comment on the items' comprehensibility, fluency, grammar, wording, perception, and the ease of completing the questionnaire. After receiving feedback and applying corrective comments, problematic items and phrases will be modified, and the research team will reread the questionnaires.

The quantitative face validity of the questionnaire will be assessed by calculating the impact of the item. For this purpose, a 5‐point Likert scale checklist will be provided to 15 experts and 15 ART candidates. This checklist includes the options ‘totally important (5),’ ‘somewhat important (4),’ ‘moderately important (3),’ ‘slightly important (2),’ and ‘not important at all (1).’ Afterward, the multiplication of the relative frequency of the participants who gave 4 and 5 points to the intended item by the mean score given by all the participants to that item will be considered as the item effect. The criteria for maintaining the subject will be a score above 1.5.

#### 2.3.3 Content Validity

The content validity of the questionnaire will be investigated quantitatively and qualitatively. Regarding qualitative content validity, comprehensiveness, compliance with Persian grammar, and appropriate and accurate use of words and phrases will be evaluated. To this end, the questionnaire will be provided to 15 experts, who will be requested to provide their written comprehensive corrective opinions.

Quantitative content validity is evaluated using the Content Validity Ratio (CVR) and Content Validity Index (CVI). To determine CVR, experts will be requested to determine the necessity of each option based on a 3‐point Likert scale, including ‘necessary (3),’ ‘Useful but not necessary (2),’ and ‘Not important or necessary (1)’. To calculate the content validity ratio, the Lawshe formula will be used [22]. Therefore, based on the points collected and the following formula, the content validity ratio will be calculated for each item. The numerical value of CVR will be calculated using the formula (Ne‐N/2)/(N/2) [23].

In this formula, Ne is the number of experts who evaluate the item as ‘Necessary,’ and N is the number of experts [24]. If more than half of the experts consider the item as ‘Essential and useful,’ that item has at least some content validity. The higher the level of experts' consensus regarding the necessity of an item, the higher the level of content validity.

To calculate CVI using a checklist on a 4‐point Likert scale, including ‘Irrelevant (1),’ ‘Moderately relevant (2),’ ‘Relevant (3),’ and ‘Totally relevant (4),’ experts' opinions on the relevance of the items to the subject under investigation will be collected. In addition, the CVI score will be calculated using the formula (N/A (N‐A)) N/2. In this formula, N is the number of expert evaluators, and A is the number of experts who will choose the ‘Irrelevant’ and ‘Totally relevant’ options. In this assessment, the item retention criterion is the CVI higher than 0.79. Moreover, items with a CVI between 0.79 and 0.70 will be revised and modified, and items with a score of less than 0.70 will be removed.

#### 2.3.4 Questionnaire Reliability

Internal consistency and stability will be determined to assess the questionnaire's reliability. To this end, a guided study will be conducted on 30 infertile couples seeking ART who are eligible for the study, and completion of the initial version of the questionnaire. Moreover, the internal consistency will be evaluated by calculating Cronbach's alpha coefficient. The accepted coefficient for consistency will be equal to or higher than 0.70. The questionnaire's reliability will be measured by repeating the completion of the questionnaire by the participants in 3 weeks and determining the ICC repeatability coefficient.

### 2.4 Third Phase: Cross‐Sectional Survey Study (Explanatory Analysis)

This phase of the study will be conducted through a cross‐sectional approach in infertile couples requesting ART referred to two private and public infertility centers in Isfahan, Iran.

#### 2.4.1 Sampling

Sampling will be conducted using the convenient method. Participants will be recruited from couples who have not entered the ovulation induction phase. The inclusion criteria for infertile couples participating in the research include having Iranian nationality, not suffering from severe mental illnesses (such as severe depression and bipolar disorder, according to the opinion of a psychiatric expert and case documents), not using donated gametes, not using a surrogate mother, and not having children. Inclusion criteria will be evaluated through an initial interview with individuals and referring to their files. The number of individuals for construct validity evaluation will be calculated based on the five participants per item ratio [25].

After explaining the study objectives and obtaining informed consent, the background information questionnaire will be completed, and the questionnaire for the psycho‐social burden of infertility will be provided to the individuals for completion as a self‐report. Regarding participants who are not literate, the questionnaire will be completed by one of the researchers as questions and answers.

At this stage, in addition to the infertility psycho‐social burden, the general health of the participants using the 28‐item general health questionnaire (28‐items GHQ) will also be completed by the last by the participants. This questionnaire measures four subscales of physical symptoms, anxiety and insomnia, social dysfunction, and depression on a 4‐point Likert scale (0–3). A higher score indicates lower health in each subscale [26]. The scale's validity and reliability to be used in Iranian hospitals have been assessed and confirmed with a Cronbach's alpha above 0.74 for all items, indicating acceptable internal reliability for both men and women [27]. Since this scale can measure the mentality of couples regarding various dimensions of health, it is a suitable scale for evaluating the criterion validity of the psychological burden scale.

#### 2.4.2 Statistics Analysis

SPSS software version 19 and plug‐in application PROCESS macro v 3.4 by Hayes. will be used to analyze the data at this stage. The Principal Component Analysis (PCA) method with orthogonal rotation of Varimax type will be used to evaluate construct validity and factor extraction. A factor loads of 0.4 and higher will be considered for the existence of agency in each factor. The Bartlett's and Kaiser‐Meyer‐Elkin (KMO) tests and Bartlett's sphericity test will be evaluated to determine the sample adequacy, and KMO above 0.9 and Bartlett's sphericity test with a significant level of less than 0.5 will be considered as a criterion of sample adequacy for factor analysis. Eigenvalue and Scree plot will also be used to determine the number of factors.

The linear regression will be used to investigate the relationship between the psycho‐social burden components and general health domains. Also, using PROCESS macro application, the direct and indirect effects of each of the psycho‐social components on general health will be evaluated. Based on the results the conceptual model will be designed.

### 2.5 Fourth Phase: Cross‐Sectional Study (Confirmatory Analysis)

In a second cross‐sectional study, the confirmatory analysis of the conceptual model, will be performed. This phase will be carried out in the third phase following the scale's psychometrics. The fit of the conceptual model developed based on the results of the third phase and using structural equation analysis will be evaluated. Since it is recommended to conduct a confirmatory analysis of the new participants' data, this phase will be conducted in the fourth phase, after the construct validity [28]. Convenience sampling will be performed among eligible couples. The research community in this study phase will be the same community that will enter the study independently. Taking into account ethical considerations, participants will complete a psychometric scale and a general health questionnaire after going through the invitation process.

The sample size will be considered with 95% confidence level and test power of 80% and at least 150 couples. In this phase the designed model (based on the results of the third phase) confirmatory factor analysis will be performed using analysis of moment structures (AMOS) software version 19. A significance level greater than 0.5 and a Chi‐square/df fit index between 0.3 and 0.6 will be considered the criterion for accepting the fit. A significance level greater than 0.5 and a Chi‐square/df fit index between 0.3 and 0.6 will be considered the criterion for accepting the fit.

## 3 Discussion

The aim of the present study is to design a conceptual model of infertility psycho‐social burden in couples who are candidates for ART. Infertility affects various aspects of individuals' lives due to multiple and wide‐ranging complications and influences the involved couples' mental health.

In many societies where having children is considered an imperative goal for couples, infertility will be associated with social problems affecting individuals' psyche since the termination of married life will be a crucial concern for couples. In addition, in societies such as Iran, having children is considered a social obligation for couples, and, therefore, infertility is associated with significant psycho‐social pressure since the couple's inability to fulfill this social commitment not only spoils the couple's privacy but also increases the social pressure to initiate the treatment process. There are studies indicating that the motive for treatment in infertile couples in these societies was mainly to dispense with the social pressure by their associates to have children [29, 30].

Although assisted reproductive treatments using advanced technologies have been associated with continuous success, the possibility of treatment failure in each treatment cycle imposes a high psychotherapeutic burden on many couples due to the expenses and complications of diagnosis and treatment procedures [31].

Additionally, addressing infertility is a critical component of sexual health and reproductive rights [32], and adopting a holistic approach to health involves taking into account issues that go beyond simple access to biomedical interventions [33]. Therefore, providing reproductive assistance services requires identifying sources of crisis for infertile couples by managing which comprehensive capacities can be provided for them. Developing a specific psychosocial conceptual model with the required validity can help identify and comprehensively plan for infertility services.

Given strong regional pronatalism, high rates of infertility, socio‐cultural prohibitions against adoption, ART has been embraced across the Middle Eastern region. Although cultural, social, and religious considerations in Iran and most countries in the Middle East have paved the way for the use of assisted reproductive treatments [34], many couples undergoing ART still endure high psychosocial pressures. Therefore, identifying the social sources imposing infertility psychological burden and the relationships between diverse psychosocial factors as a conceptual model helps design psychological interventions since using a conceptual model is a strategic principle for developing effective health interventions [35]. Furthermore, a valid scale for evaluating the psychosocial burden of infertility is the result of the present study that can be used by psychology and medical sociology researchers. This scale will also be a reliable tool for psychologists, psychoanalysts, and social workers in infertility centers to identify the source of psychological burden in infertile couples.

In developing the protocol for design the conceptual model of the infertility psycho‐social burden, efforts have been made to design a document‐based model based on the specific cultural and social conditions of Iranian society; however, it should be considered that using the results of this study can be associated with limitations. While efforts are made to recruit participants with extensive experience of the psychosocial burden of infertility and participants with appropriate diversity, those who decline may have experienced this burden more profoundly than others. Therefore, this issue is considered a limitation of the study.

The second limitation of this study will be the application of the model in populations with cultures different from the Iranian population. Furthermore, its utilization in other societies needs to be evaluated. In conclusion, the psycho‐social burden of infertility questionnaire developed based on this study protocol will undergo various psychometric stages, and its results can be reliable for researchers. Moreover, the prepared questionnaire can be used to evaluate the psycho‐social burden of infertile couples to plan for fertility services for infertile couples at the national level. But, the generalizability of this study's results to communities with cultures and social conditions different from those of Iran is limited.

## Author Contributions

**Shekofeh Maleki:** conceptualization, writing – original draft, methodology, formal analysis, data curation, writing – review and editing, visualization, investigation. **Ashraf Kazemi:** conceptualization, investigation, funding acquisition, writing – original draft, methodology, writing – review and editing, software, formal analysis, supervision, project administration. **Hamid Nasiri‐Dehsorkhi:** conceptualization, methodology, writing – original draft, writing – review and editing, supervision. **Nafisehsadat Nekuei:** conceptualization, methodology, writing – original draft, writing – review and editing, supervision, formal analysis.

## Ethics Statement

Ethical approval for this study has been obtained by the ethics committee affiliated with Isfahan University of Medical Sciences, Isfahan, Iran. (IR. MUI. NUREMA. REC.1402.127). All the procedures to the participants will be in accordance with the ethical standards of the Isfahan University of Medical Sciences.

## Consent

Informed consent will be obtained from all participants.

## Conflicts of Interest

The authors declare no conflicts of interest.

### 1 Transparency Statement

The lead author Ashraf Kazemi affirms that this manuscript is an honest, accurate, and transparent account of the study being reported; that no important aspects of the study have been omitted; and that any discrepancies from the study as planned (and, if relevant, registered) have been explained.

## Data Availability

The data that will be supported the findings of this study awill be available from the corresponding author upon reasonable request.
